# A Hadamard transform UV absorption detection for high performance liquid chromatography. Part I. Preliminary experiments

**DOI:** 10.1155/S1463924695000137

**Published:** 1995

**Authors:** J. G. Brayan, D. J. Malcolme-Lawes, C. D. Mew, S. Xie

**Affiliations:** Centre for Research in Analytical Chemistry and Instrumentation King's College London Strand London WC2R 2LS UK

## Abstract

The principles and design of a Hadamard transform UV absorbance detector for liquid chromatography are outlined, and some spectra of aromatic compounds passing through its flow cell are presented. This approach could be valuable in providing a low-cost multi-wavelength detection method for liquid chromatography.


Journal of Automatic Chemistry, Vol. 17, No. 2 (March-April 1995), pp. 77-82

### A Hadamard transform UV absorption detection for high performance liquid chromatography. Part I. Preliminary experiments

J. G. Brayan, D. J. Malcolme-Lawes, C. D. Mew and S. Xie

*Centre for Research in Analytical Chemistry and Instrumentation, King's College London, Strand, London WC2R 2LS*

*The principles and design of a Hadamard transform UV absorbance detector for liquid chromatography are outlined, and some spectra of aromatic compounds passing through its flow cell are presented. This approach could be valuable in providing a low-cost multi-wavelength detection method for liquid chromatography.*

### Introduction

Most of the UV-visible spectrophotometers used as detectors for high performance liquid chromatography are single or multiple wavelength models. These instruments are capable of monitoring absorbance changes at one wavelength at a time. Peak monitoring at several wavelengths can provide additional information which is particularly useful when a chromatogram contains unidentified or unresolved peaks. A more detailed discussion of multiple wavelength chromatographic detection is provided elsewhere [1-3].

Variable wavelength absorption detectors typically contain a monochromator which is used to isolate the wavelength of interest. Polychromatic radiation from a source such as a deuterium lamp is collimated and directed onto a diffraction grating where it is dispersed. Radiation of one wavelength is then focused onto the sample cell and the reduction in light intensity due to absorption by the sample can be measured by an optical transducer located on the other side of the cell. The wavelength of the radiation that passes through the cell can be altered by changing the angle at which the incident radiation strikes the grating.

Sequential multiple wavelength analysis can be achieved by rapid scanning [1, 4, 5 and 6]. This involves rapidly changing the wavelength of the light that passes through the sample cell so that the absorbance at a range of wavelengths can be measured before the sample leaves the cell. Multiple wavelength detection can also be carried out using photodiode array detectors [1, 7, 8]. These instruments make use of reverse optics, where polychromatic light is initially passed through the sample cell before being dispersed by a diffraction grating. An array of photodiodes is then positioned so that each element will accept radiation of one narrow wavelength range. These instruments allow true simultaneous multi-wavelength detection, but they are much more expensive than single or variable wavelength detectors.

The transducer elements in both rapid scanning and photodiode array detectors are positioned to receive radiation within a narrow band of wavelengths. The light intensity reaching the transducer would be drastically increased if light with many different wavelengths could be measured simultaneously. When a series of readings are taken in which light with an encoded group of wavelength combination is allowed to reach a detector, a mathematical decoding technique can be applied to resolve the signals into their individual components. This process is known as multiplexing [9-12]. There are two types of mutiplexing: the first makes use of interference effects for wavelength encoding and Fourier transforms for resolution; the second uses masks and discrete transforms, such as the Hadamard transform.

One of the most straightforward methods of wavelength encoding makes use of the Hadamard matrix [13]. Hadamard transform instruments have a moving mask in front of the optical transducer which allows certain wavelengths through to the detector at a particular time. The calculations required for the Hadamard transform can be carried out quite rapidly by a personal microcomputer (PC), which can also be used for the control of the mask and data collection.

The usual purpose of multiplexing is to gain an improvement in sensitivity over single wavelength instruments. The sensitivity of a spectrometer is limited by the component that contributes the maximum amount of noise to the system. In detector-noise-limited cases, multiplexing can lead to improved signal to noise ratios because the amount of radiation reaching the detector is increased, but the noise level remains constant. This is known as the Fellget advantage [9-12], and it is this advantage which can be obtained in Fourier transform infra-red spectroscopy.

A light source, such as a deuterium lamp, will generally contribute a greater level of noise to the system than a detector such as the photomultiplier tube or photodiode. So most spectrometers that operate in the UV range are source limited. Under such circumstances the Fellget advantage is not realized because the noise reaching the detector increases with the size of the signal. Theoretical [14] and practical [15] results have suggested that multiplexing will lead to a degradation of the signal to noise ratio for broad band spectra in the UV region. Consequently, multiplex methods have not been widely used in UV visible spectroscopy.

0142-0453/95 $10.00 © 1995 Taylor & Francis Ltd.

However, there are instances where the detector noise is significant. For example, in liquid chromatography the aim in UV absorbance detector design is to use a flow cell with a small volume and a reasonable light path length, so the amount of light transmitted through the cell can be small. Furthermore, in the UV region, Hadamard transform spectroscopy may offer a simplified method of multiple wavelength detection for HPLC. The mechanics of such a system would be simpler than that use in conventional rapid scanning, and it would not rely on a complex and expensive optical system like the diode array.

### *Hadamard transform spectroscopy*

The Hadamard transform can be best described in terms of a weighing analogy [9, 11]. If three items, A, B and C are to be weighed, the most obvious strategy would be to weigh them one at a time in three separate measurements.

An alternative approach would be to carry out three separate determinations, but to weigh two of the items each time, as shown in equations (1)–(3):

$$X = A + B \quad (1)$$

$$Y = A + C \quad (2)$$

$$Z = B + C \quad (3)$$

The values of A, B and C can be determined from $X$, $Y$ and $Z$ using equations (4)–(6):

$$A = \tfrac{1}{2}(X + Y - Z) \quad (4)$$

$$B = \tfrac{1}{2}(X + Z - Y) \quad (5)$$

$$C = \tfrac{1}{2}(Y + Z - X) \quad (6)$$

Equations (1)–(3) can be rewritten using a 3 × 3 matrix, as shown in equation (7):

$$\begin{bmatrix} X \\ Y \\ Z \end{bmatrix} = \frac{1}{2}\begin{bmatrix} 1 & 1 & 0 \\ 1 & 0 & 1 \\ 0 & 1 & 1 \end{bmatrix} \times \begin{bmatrix} A \\ B \\ C \end{bmatrix} \quad (7)$$

By obtaining measurements for $X$, $Y$ and $Z$ the values for A, B and C can be obtained by multiplying the vector containing $X$, $Y$ and $Z$ by the inverse of the matrix as shown in equation 8:

$$\begin{bmatrix} A \\ B \\ C \end{bmatrix} = \frac{1}{2}\begin{bmatrix} 1 & 1 & -1 \\ 1 & -1 & 1 \\ -1 & 1 & 1 \end{bmatrix} \times \begin{bmatrix} X \\ Y \\ Z \end{bmatrix} \quad (8)$$

The matrix shown in equation (7) is of the type known as an $S$-matrix, which was originally defined by Hadamard [13]. The mathematical definition and methods of construction of $S$-matrices have been described by Harwit and Sloane [10].

The $S$-matrix transform can be applied to spectroscopy, where the intensity of radiation with wavelengths selected according to an $S$-matrix can be simultaneously measured and the results multiplied by the inverse of the $S$-matrix to obtain the intensities resulting from each of the individual wavelengths.

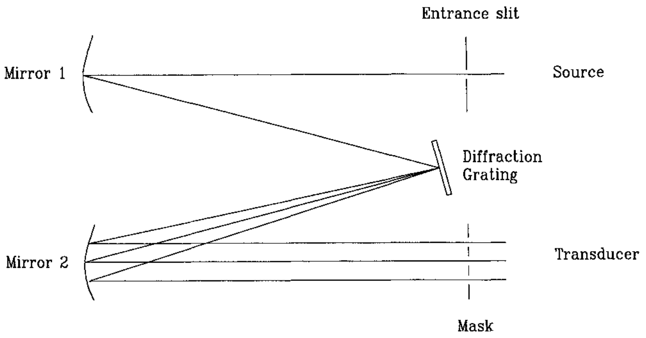

*Figure 1. Optical arrangement for a Hadamard transform spectrometer.*

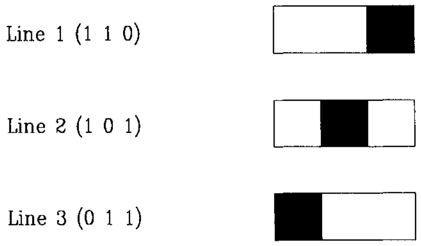

*Figure 2. A Hadamard mask based on a 3 × 3 S-matrix.*

The optical arrangement for a Hadamard transform spectrometer is identical to that use for a conventional spectrometer with the exception that a moving mask is placed at the exit plane rather than a single slit. Figure 1 shows an example of such an arrangement based on the Czerny–Turner monochromator.

A mask based on the matrix in equation 7 is shown in figure 2. To determine the intensities of light with three different wavelengths (A–C), the light would be initially dispersed by the spectrometer and focused onto the first line of the mask. This allows light with wavelengths A and B, but not C, to reach the transducer. After the first reading ($X$) is taken, the mask would be moved so that the light fell onto the second line. A second reading ($Y$) would be taken and the process repeated for the line ($Z$). The intensities (A–C) could be determined by the multiplication of the inverse matrix by the three readings ($X$–$Z$) as shown in equation (8).

There are likely to be more than three wavelengths of interest in most circumstances, so larger $S$-matrices need to be found. A matrix in which each line is equal to the previous line, shifted one place to the left, with the number from the left side being moved to the right, is known as a left circulant matrix. This type of matrix is particularly useful in spectroscopy, since a single line mask can be constructed which is simply shifted one place to the left each time a reading is taken. The first line of a left circulant $S$-matrix can be generated using maximal length shift register sequences [10]. Using this method, it is possible to produce $n \times n$ $S$-matrices, where $n = 2^m - 1$ and $m$ is any whole number.

The design of the authors' computer controlled Hadamard transform detector is illustrated in figure 1 and described below. It was based on a mask constructed using a 63 × 63 S-matrix at the exit slit. Some spectra of aromatic compounds within the flow cell were recorded and the linearity of the recorded absorbance with sample concentration was examined.

### Experimental

### *Instrumentation and materials*

Molecular absorption spectra were collected using a Perkin–Elmer 552 double beam scanning spectrophotometer (Perkin–Elmer, Buckingham, UK). The chromatograph consisted of an ACS (Macclesfield, Cheshire, UK) model 351 solvent delivery system, a Rheodyne (Cotati, California, USA) model 7125 injection valve with a 20 μl injection loop and a Spherisorb ODS1 reversed phase column (Phase Separations, Queensferry, Clwyd, UK).

The mobile phases were prepared from HPLC grade methanol and water (Rathburn Chemicals, Walkerburn, Scotland, UK). The compounds used for analysis included the polyaromatic hydrocarbons, phenanthrene and 1-Naphthol (BDH Chemicals Ltd, Poole, UK), pyrene (Aldrich Chemical Co. Ltd, Gillingham, UK), carbazole (Hopkin & Williams Ltd., Chadwell Heath, UK) and trans-stilbene (Koch-Light Laboratories, Colnbrook, UK).

### *The Hadamard detector system*

A deuterium lamp (Cathodeon, Cambridge, UK) was used to provide a continuous source of radiation with a wavelength range at the mask of 207–381 nm. A front surface coated concave mirror with a focal length of 50 mm was used to focus the light onto the centre of a 13·5 μl flow cell (ACS Ltd, Macclesfield, UK). The flow cell was placed at the entrance of the spectrometer. The light that emerged from the cell was collimated using a spherical mirror with a focal length of 100 mm and directed onto a 1500 grooves per mm diffraction grating (American Holographic, Littleton, Massachusetts, USA). The dispersed light was then focused onto the Hadamard mask using a mirror with a focal length of 125 mm. The light intensity was measured using a photomultiplier tube (Thorn-EMI Ltd, Ruislip, UK, type 9804QB) mounted behind the mask. The entire arrangement was housed within a 2 feet × 1 foot × 1 foot wooden box.

The Hadamard mask was based on a 63 × 63 left circulant S-matrix. The first line of the matrix which is shown below, was generated using a computer program based on maximal length shift register sequences [10]:

00000100001100010100111101000111
0010010110111011001101010111111

A one in the matrix represents a slit in the mask which allows light with a particular wavelength range to pass to the transducer; there is no opening when a zero appears. Each line of the matrix was reproduced in the mask as a series of slits 2 mm in height and 0·5 mm wide; an example is shown in figure 3(*a*). Thus the total width of the mask was 63 × 0·5 mm = 31·5 mm. The 63 lines of the mask were arranged on a circular metal disk with a diameter of 134 mm. The mask was rotated to move from one line of the matrix to the next. A 64th line consisting of a completely open slot was added to aid in mask alignment and so that the mask pattern would be symmetrical. The entire mask is shown in figure 3(*b*). The hole in the outer edge of the wheel, alongside the 'open' slot, was also used for alignment using an optical switch (although this was not employed in the experiments described below). The mask was fabricated from diagrams such as those in figure 3 by The Old Powerhouse Engineering Co (London, UK), using electro-forming techniques.

The mask was rotated using a belt-drive system connected to a stepping motor with a step angle of 1·8° (RS Components Ltd, Corby, UK). As a mask consisted of 64 lines, a step angle of 5·625° was required to move from one line to the next. A pulley with a diameter of 19·35 mm and 25 teeth was mounted on the motor, and a pulley with a diameter of 24·95 mm and 32 teeth was used on the wheel. Thus four 1·8° steps of the motor caused the wheel to move 5·625° in total.

### *System control*

Data collection and stepping motor control was carried out using a Compaq 386 microcomputer. Analogue to digital conversion of the signal from the photomultiplier tube was achieved using a CIL multifunction interface card (CIL Group, Lancing, UK), later changed to a Semaphore PCL-816 interface (Semaphore Cards, London, UK). Step signals were sent to the motor using a home-made interface card. This card was also used to receive signals from an optical switch.

The mask was moved and data collected at the appropriate times by a program written in Microsoft QuickBasic; an assembly language routine was also used in an attempt to increase the step speed and rate of data collection. After each rotation of the wheel 63 intensity readings were obtained which were converted to the intensity values for each wavelength using a program based on the fast S-matrix transform described by Harwit and Sloane [10].

As the instrument is only a single channel device, it was necessary to take two sets of intensity readings in order to determine the absorption spectra. A set of readings was taken while the wheel was rotated once with only methanol present in the cell. A solution of analyte in methanol was then pumped into the cell and a second set of readings was collected. The intensity of light at each wavelength was calculated using the Hadamard transform in each case and the absorbance readings were recorded. For chromatographic measurements, the wheel was rotated continuously after the injection of the sample. The readings taken during the first rotation were used to calculate the intensities for eluent alone, and subsequent readings were used to obtain absorbance values relative to the eluent.

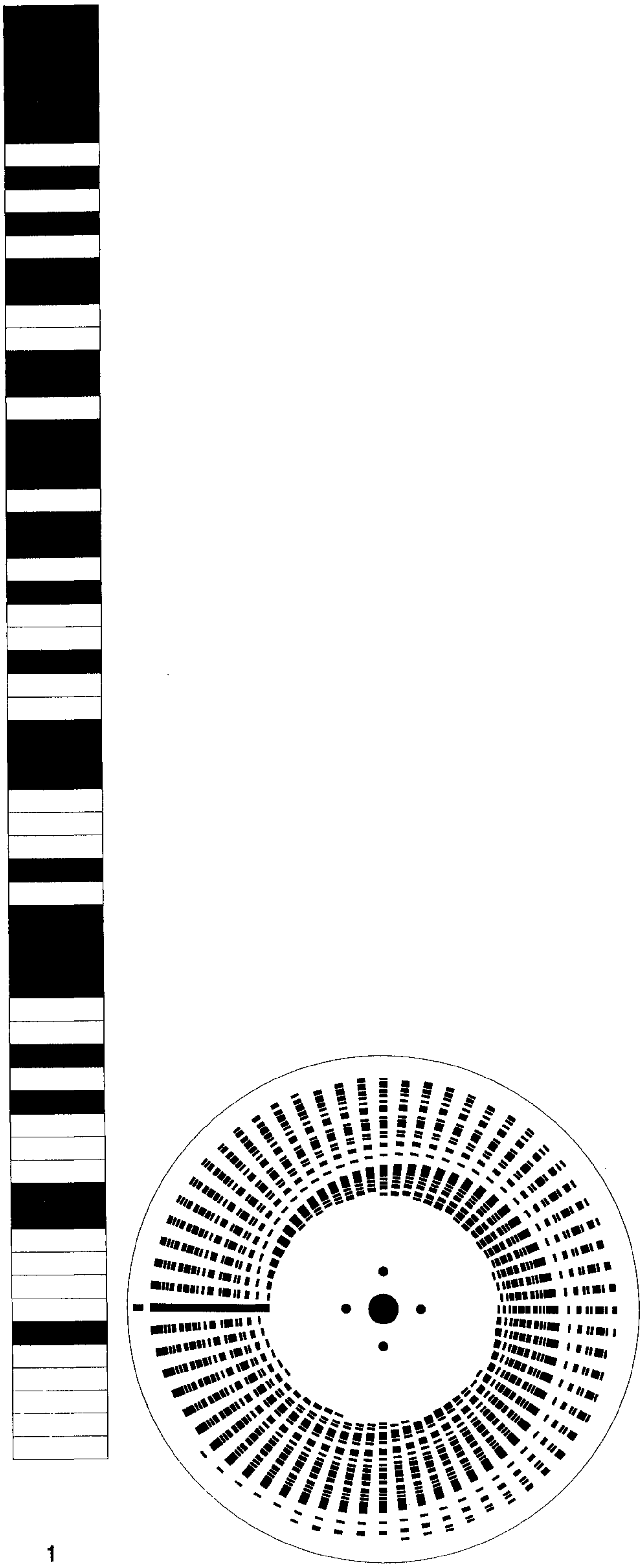

(a) (b)

*Figure 3. The mask for the Hadamard spectrometer based on a 63 × 63 S-matrix. (a) A single mask element; (b) the complete 63 element mask. The black marks represent open slits which allow light to pass to the transducer, and correspond to ones in the matrix.*

*Wavelength calibration*

The performance of a particular diffraction grating is given by the grating equation (equation (9)):

$$\sin i + \sin \theta = m\lambda/d \qquad (9)$$

$i$ is the angle at which light strikes the grating and $\theta$ is the angle of diffraction of radiation with wavelength $\lambda$, $m$ is the order of diffraction and $d$ is the distance between the grooves on the grating.

A measure of the ability of a particular spectrometer system to separate radiation into its component wavelengths may be obtained by calculating the reciprocal linear dispersion (equation (10)):

$$R_D = d \cos \theta / f \qquad (10)$$

$R_D$ has units of wavelength per unit length in the focal plane, and $f$ is the focal length of the focusing mirror. At 254 nm, the spectrometer was calculated to have a reciprocal linear dispersion of 5·24 nm/mm. This figure will not change much for most wavelengths in the UV range since $\cos \theta$ is always close to 1. Therefore if the width of the mask at the focal plane is 31·5 mm, it should be possible to observe a wavelength range of 165 nm.

The spectrometer was calibrated by placing line filters in front of the flow cell, so that only light with a particular wavelength was allowed to enter the system. When the data were collected, and the Hadamard transform carried out, a line appeared at the position corresponding to the wavelength of the radiation passing through the spectrometer. The results indicated that each slit covered a range of 2·8 nm and that the range that could be observed by the instrument is 207–381 nm. This agreed well with the range estimated using equations (9) and (10). In operation, the mask was rotated at approximately 2 revolutions per second.

### Results and discussion

An example of the actual intensities recorded through the 63 different mask slots are shown in figure 4(a) (there is methanol only in the flow cell), and a base-line absorbance spectrum (i.e. methanol only in the flow cell) is shown in figure 4(b). Absorption spectra for five different aromatic compounds were recorded and the results, which closely resembled those collected with a commercial scanning spectrophotometer, are shown in figure 5. Table 1 shows the principal absorption bands recorded using the Hadamard transform detector, compared with those from a commercial spectrometer using a conventional cuvette. Clearly the agreement is satisfactory, bearing in mind the limited resolution resulting from the use of a 63 element mask (i.e. 2·8 nm per slot).

The linearity of response as a function of sample concentration was determined by recording spectra from several samples of pyrene in methanol. The results are shown in figure 6, and indicate that the system gives excellent linearity over the absorbance range.

The results show that the detector can generate spectra in approximately 0·5 s from a sample within a 13·5 µl flow-cell, which are in good agreement with those

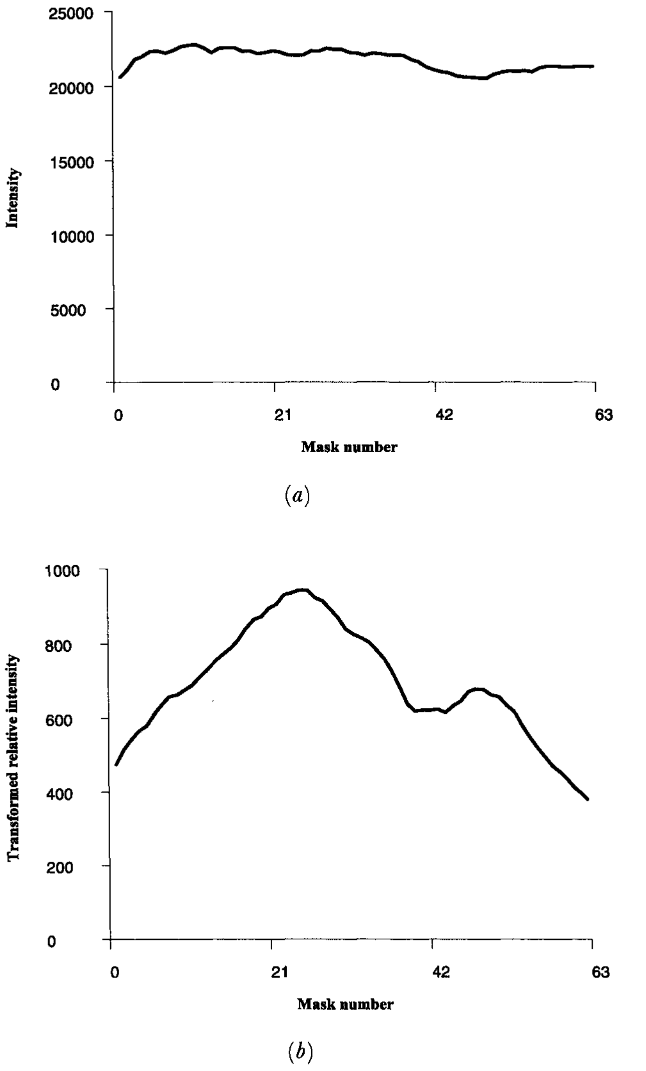

*Figure 4. Results obtained with methanol in the flow cell. (a) Intensity measurements recorded through each mask element; (b) the transformed intensity spectrum.*

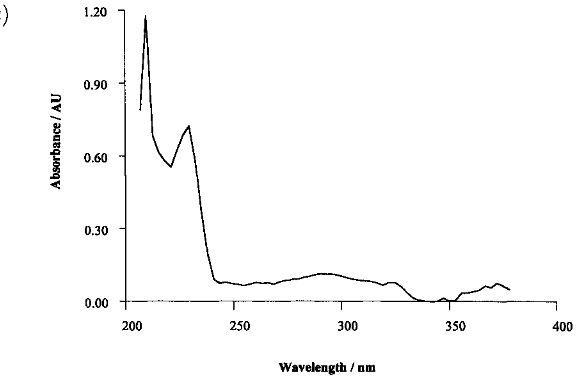

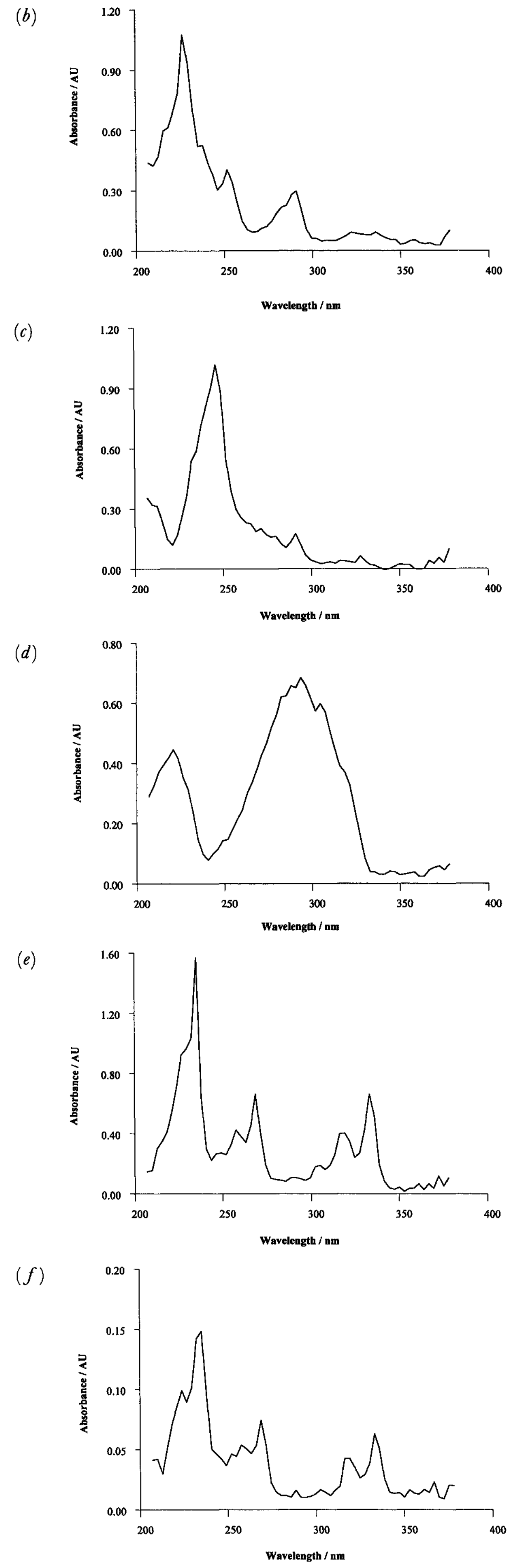

*Figure 5. UV absorption spectra of samples in methanol solvent, collected using Hadamard transform spectroscopy. (a) 5 ppm of 1-naphthol; (b) 5·0 ppm of carbazole; (c) 5·0 ppm of phenanthrene; (d) 5·0 ppm of trans-stilbene; (e) 5·0 ppm of pyrene; (f) 0·5 ppm of pyrene.*

*Table 1. The principal absorption bands recorded for a number of samples.*

| Analyte | Hadamard transform spectrometer (±2·8 mm) | Commercial scanning spectrometer |
|---|---|---|
| Trans-stilbene | 224, 297, 308 | 226, 295, 306 |
| Pyrene | 229, 238, 260, 271, 336 | 230, 238, 260, 271, 333 |
| 1-Naphthol | 230, 296 | 232, 296 |
| Phenanthrene | 248, 272, 291 | 249, 271, 291 |
| Carbazole | 230, 255, 294 | 233, 255, 292 |

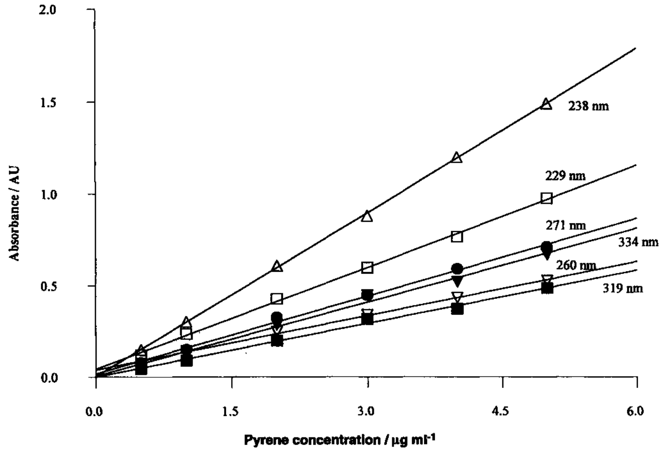

*Figure 6. Peak height (absorbance units) plotted against concentration (µg/ml) for a series of pyrene standards.*

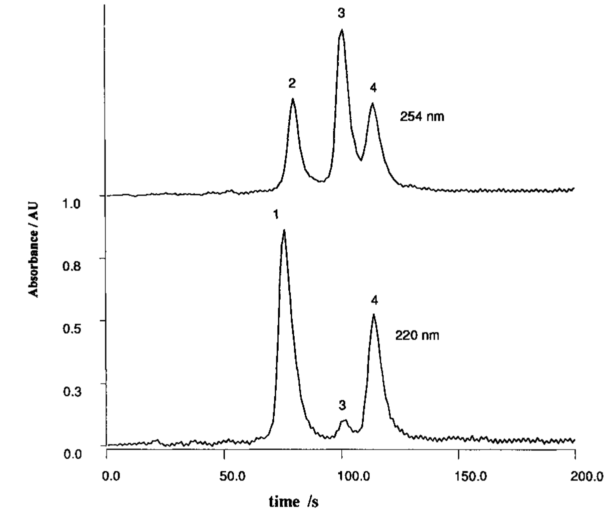

*Figure 7. A chromatogram recorded at 220 and 254 nm from a sample containing (1) naphthalene (20 ppm), (2) biphenyl (20 ppm), (3) anthracene (20 ppm) and (4) fluoranthene (40 ppm), eluted from an ODS column using an eluent of 90% methanol/10% water.*

obtained from a commercial spectrometer using a much larger sample and taking approximately 3 min each to scan. These findings indicate that the approach may be valuable in providing a low-cost multi-wavelength detection method for liquid chromatography and flow injection analysis, where speed and cell size are of importance.

An example of a chromatogram recorded with the system is shown in figure 7. The chromatogram was recorded at 220 and 254 nm for a four component mixture. Peaks 1 and 2 (Naphthalene and Biphenyl) almost co-elute, but have distinctly different absorbances, while peak 3 (Anthracene) absorbs strongly at 254 nm but much less so at 220 nm. The last peak (Fluoranthene) absorbs at both wavelengths.

### Summary

The basic principles and the construction of a Hadamard transform detector have been described, and the spectral results obtained from a number of aromatic compounds presented. The results show that the detector can generate spectra from a 13·5 µl flow-cell, which are in good agreement with those obtained from a commercial spectrometer using a much larger sample, and within a time period of about 0·5 s. These findings indicate that the approach may be valuable in providing a low-cost multi-wavelength detection method for liquid chromatography.

### References

1. Yeung, E. S. (Ed.), *Detectors for Liquid Chromatography.* John Wiley and Sons, New York (1986).
2. Scott, R. P. W., *Liquid Chromatography Detectors.* Elsevier, Amsterdam (1986).
3. Vickrey, T. M. (Ed.), *Liquid Chromatography Detectors.* Marcel Dekker Inc., New York (1983).
4. Saitoh, K. and Suziki, N., *Analytical Chemistry*, **51** (1979), 1683.
5. Denton, M. S., DeAngelis, T. P., Yacynych, A. M., Hieneman, W. R. and Gilbert, T. W., *Analytical Chemistry*, **48** (1976), 20.
6. Baram, G. I., Grachev, M. A., Komarova, M. I., Perelroyzen, M. P., Bolvanov, Yu. A., Kuzman, S. V., Kargeltsev, V. V. and Kuper, E. A., *Journal of Chromatography*, **264** (1983), 69.
7. Miller, J. C., George, S. A. and Willis, B. G., *Science*, **218** (1982), 241.
8. George, S. A. and Maute, A., *Chromatographia*, **15** (1982), 419.
9. Busch, K. W. and Busch, M. A., *Multielement Detection Systems for Spectrochemical Analysis.* John Wiley and Sons, New York (1990).
10. Harwit, M. and Sloane, N. J. A., *Hadamard Transform Optics.* Academic Press, New York (1979).
11. Marshall, A. G. (Ed.), *Fourier, Hadamard and Hilbert Transformations in Chemistry.* Plenum Publishing Corp., New York (1982).
12. Vanassee, G. A. (Ed.), *Spectrometric Techniques.* Academic Press, New York (1977).
13. Hadamard, J., *Bull. Sci. Math.*, **17** (1893), 240.
14. Larson, N. M., Crosmun, R. and Talmi, Y., *Applied Optics*, **13** (1974), 2662.
15. Plankey, F. W., Glen, T. H., Hart, L. P. and Winefordner, J. D., *Analytical Chemistry*, **46** (1974), 1000.

